# Post-COVID-19 Rehabilitation: Perception and Experience of Austrian Physiotherapists and Physiotherapy Students

**DOI:** 10.3390/ijerph18168730

**Published:** 2021-08-18

**Authors:** Barbara Scheiber, Claudia Spiegl, Claudia Wiederin, Erika Schifferegger, Natalia Schiefermeier-Mach

**Affiliations:** 1 Department of Physiotherapy, FH Gesundheit Tirol/Health University of Applied Sciences Tyrol, 6020 Innsbruck, Austria; barbara.scheiber@fhg-tirol.ac.at (B.S.); claudia.spiegl@fhg-tirol.ac.at (C.S.); claudia.wiederin@fhg-tirol.ac.at (C.W.); erika.schifferegger@fhg-tirol.ac.at (E.S.); 2 FH Gesundheit Tirol/Health University of Applied Sciences Tyrol, 6020 Innsbruck, Austria

**Keywords:** post-acute COVID-19 syndrome, long COVID-19, physical therapy specialty, respiratory therapy, survey, physical and rehabilitation medicine, education

## Abstract

The rehabilitation needs of COVID-19 survivors are increasingly recognized, with a focus on combating respiratory and neuromuscular dysfunctions. The aim here was to explore the perception of Austrian physiotherapists and physiotherapy students on post-COVID-19 rehabilitation care and to identify barriers for the application of sufficient rehabilitation. We analysed current knowledge and practical skills in respiratory physiotherapy, performing a cross-sectional national survey among physiotherapists working in outpatient settings and physiotherapy students in their last academic year of bachelor-level education in Austria. Out of 255 survey participants, one-third already had inquiries to treat post-COVID-19 patients, and the majority of respondents expected a further increased inflow of patients with rehabilitation needs (64.2%). Only 11.2% of respondents reported feeling sufficiently informed about post-COVID-19 rehabilitation. A total of 68.2% of students and up to 48.1% of physiotherapists favoured a COVID-19-specific adaptation already in the basic academic education, and 74.1% of survey participants indicated interest in attending specific training. Concerning respiratory physiotherapy, our data showed discrepancies between the estimation of the importance of specific examination and treatment techniques and the level of current experience. There is a clear lack of experience in implementing effective device-based respiratory therapy. Our data indicate an urgent need to develop new education and training programs with a focus on the interdisciplinary rehabilitation of patients with post-COVID-19 syndrome.

## 1. Introduction

An ongoing pandemic of coronavirus disease 2019 (COVID-19) caused by the SARS-CoV-2 virus strongly affects all levels of the health care system. This highly infectious respiratory disease has yet infected more than 199 million people worldwide and has already resulted in 4.2 million deaths [1]. Austria was one of the first COVID-19-affected countries in Europe with the Western Austrian province Tyrol hit by COVID-19 as a first area. Starting from two cases diagnosed in Tyrol in February 2020, the situation has rapidly deteriorated. With more than 655,000 infected persons so far, COVID-19 has become a supranational epidemic [1,2,3].

COVID-19 is a multi-organ disease with a broad spectrum of acute, subacute and long-term manifestations [4]. Symptoms of acute COVID-19 infection include cough, fever, fatigue, pneumonia and dyspnoea [5]. Severe respiratory symptoms may lead to a life-threatening respiratory failure (ARDS; acute respiratory distress syndrome), resulting in the urgent need for invasive ventilation at an intensive care unit (ICU) [6]. Patients remain bedridden in a prone position for extended periods, which can cause post-ICU dysphagia, muscle weakness, general deconditioning, myopathy and neuropathy, as well as musculoskeletal dysfunctions [7,8]. It is estimated that up to 14% of worldwide infected COVID-19 patients develop severe acute respiratory infection leading to hospitalization and ventilation [9].

Similar to previous coronavirus diseases (SARS, MERS [10,11,12,13]), hospitalized and non-hospitalized patients recovering from COVID-19 may suffer from persistent residual symptoms, including impaired pulmonary and physical function [14]. Long-term consequences of COVID-19 infection strongly decrease quality of life and cause emotional distress [15,16]. Post-acute COVID-19 syndrome, defined as “persistent symptoms and/or delayed long-term complication of SARS-CoV-2 infection beyond 4 weeks from the onset of symptoms” [14], was reported in more than one-third of individuals in the USA [17]. Further findings from China [16] and European countries (the UK [18], France [19], Italy [15] and Spain [20]) also reported persistent symptoms such as fatigue (in 35–63% of individuals), joint pain (5–27%), dyspnoea (11–43%), chest pain (5–22%) and other (reviewed in [14]).

Consequently, the emerging rehabilitation needs of individuals recovering from COVID-19 are increasingly recognized. The evidence-based role of physiotherapy interventions arose as highly relevant in addressing COVID-19 rehabilitation. Acute and post-acute rehabilitation in hospital settings and long-term rehabilitation in outpatient practices were reported to be of notable benefit for patients [21,22,23]. The COVID-19 pandemic is currently changing physiotherapeutic practice and will further strongly affect the rehabilitation sector, including patient care, education and research. Even though clinical studies on post-acute COVID-19 rehabilitation are still ongoing, several reports and guidelines provide recommendations for physiotherapeutic rehabilitation based on previous SARS/MERS experience as well as being based on recent data and patient case studies [8,24,25,26,27]. Recommended physiotherapy for post-acute COVID-19 syndrome can be conducted at home or in outpatient settings [8,9,28]. In line with international guidelines, the Austrian physiotherapy professional association *Physio Austria* has issued recommendations for physiotherapists with a focus on (i) respiratory rehabilitation, (ii) joints movement limitations and joints pain, (iii) rehabilitation of neurologic and sensorimotor deficits and (iv) training to improve general health condition, basic ADL training and electrotherapy [28]. However, previous studies revealed that the publication of guideline recommendations does not automatically lead to their uptake and change of therapeutical strategies in physiotherapy practices. Several barriers to guideline implementation were identified, including knowledge transfer, time for implementation, interest in implementation, and need for technical equipment [29,30,31].

In the context of the ongoing pandemic, the objective of our study was to analyse the current perception and experience of physiotherapists and physiotherapy students regarding evidence-based post-COVID-19 rehabilitation.

Specific goals of our study were to explore the perceptions of Austrian physiotherapists regarding the impact of COVID-19 rehabilitation care that the profession can provide, to assess interest in and demand of COVID-19 pandemic-related changes in physiotherapy education and additional training courses to enable more effective rehabilitation and to collect and analyse data about current experiences in respiratory physiotherapy, with emphasis on examination and treatment techniques as well as available equipment.

To the best of our knowledge, such a study has not been performed before. Our data revealed barriers to rapid implementation of effective post-acute COVID-19 rehabilitation into outpatient physiotherapy practice.

## 2. Materials and Methods

A cross-sectional national survey among physiotherapists working in outpatient settings and among physiotherapy students was conducted in Austria between December 2020 and February 2021.

### 2.1. Survey Development

A systematic literature search did not identify any existing instrument to investigate the implementation of effective respiratory physiotherapy for post-COVID-19 rehabilitation. We modified previously published survey questions concerning general aspects of respiratory rehabilitation [32] and further conducted face-to-face, telephone and online conversations with physiotherapy experts. The motivation behind these conversations was to compile suggestions and experiences for the course and purpose of the survey. Therefore, a customized questionnaire was designed. Before dissemination, the research team and three critical physiotherapists independent of the main sample reviewed the questionnaire content and its utility. Based on their feedback, minor changes were made to the survey instrument with regard to the wording and order of the items. To avoid an undesirable tendency toward the middle, 5-point Likert scales were adapted to 4-point scales. In addition, the option to tick “unsure” or “no experience” was provided. The final survey consisted of 40 questions divided into four sections. The sections covered socio-demographic data (2 questions), information on therapists’ education and current field of work (4 questions), COVID-19 specific background knowledge (9 questions) and self-assessment of examination and treatment experience, including the use of devices for respiratory physiotherapy (25 questions). The survey was conducted online using soscisurvey.de (SoSci Survey GmbH, Munich, Germany). The questionnaire in English is attached as Supplementary PDF 1.

### 2.2. Survey Participants

The survey recruited registered physiotherapists practicing in outpatient settings as well as physiotherapy students in their last academic year of bachelor-level education in Austria. The inclusion criteria for participation in the survey were working as a registered physiotherapist in an outpatient physiotherapy setting or being a final-year physiotherapy student in Austria. Exclusion criteria were working exclusively in an inpatient setting, practicing outside of Austria and being a physiotherapy student in his or her first or second educational year. In order to address students and alumni within Austria, all universities of applied sciences for physiotherapy education were contacted. In addition, *Physio Austria*, the Austrian professional physiotherapy association, announced the survey to its members in its regular newsletter (5336 physiotherapists in 2020) [33]. To invite the participants, a link to the online survey was provided via email and distributed via social and private networks. A reminder was sent out 4 weeks after the first invitation if contacted participants had not answered.

### 2.3. Ethics

Approval for the study was granted by the Private University for Health Sciences, Medical Informatics and Technology, and the Research Committee for Scientific Ethical Questions (RCSEQ, Hall in Tirol, Austria, Number 2834). All respondents participated anonymously and voluntarily. Completion and submission of the online survey represented an informed consent of the participants, as described in the introduction of the questionnaire.

### 2.4. Data Analysis

Data were analysed using IBM SPSS version 26.0 statistical software (IBM SPSS Inc., Chicago, IL, USA), and graphs were prepared using PRISM software (GraphPad Software, San Diego, CA, USA). Descriptive analyses were used to present the current situation regarding the perception and experience of Austrian physiotherapists and physiotherapy students in post-COVID-19 rehabilitation. Questions with closed answer formats were analysed descriptively using frequencies and percentages. Evaluation of all available cases, including incomplete data sets, did not reveal conspicuously high numbers of dropouts for any of the questions. Original data, including a number of answers and percentages, are summarized in Supplementary PDF 2 and Supplementary Tables S1–S3. Responses to open-ended questions were transcribed verbatim. Two researchers performed a simple content analysis to identify recurring entries and to build categories.

## 3. Results

### 3.1. Response Rates

The survey link was accessed 821 times (survey views). The first survey question was completed 308 times (37.5% of survey views). A total of 255 surveys provided relevant data and were analysed for this project (82.8% of first survey page completion, 31.1% of survey views).

### 3.2. Participants Characteristics

Out of 255 respondents, 80.8% were physiotherapists, and 19.2% were physiotherapy students in their last educational year. Respondents indicated a median age of 32.0, with an interquartile range (IQR) of 26–40 and a median work experience of 7.0 (IQR 1–15) years, ranging from less than 1 year to 38 years of experience. Physiotherapists and physiotherapy students from all federal states of Austria participated in the study, 68.6% of therapists were working in independent physiotherapy practices. With regard to gender distribution, the data of our survey represent the actual ratio in the physiotherapy profession in Austria (75% female; 25% male) [34]. Respondents’ educational and work field characteristics are presented in Table 1.

### 3.3. Future Impact of COVID-19 on Physiotherapy Academic Education and Profession

**Figure 1:**
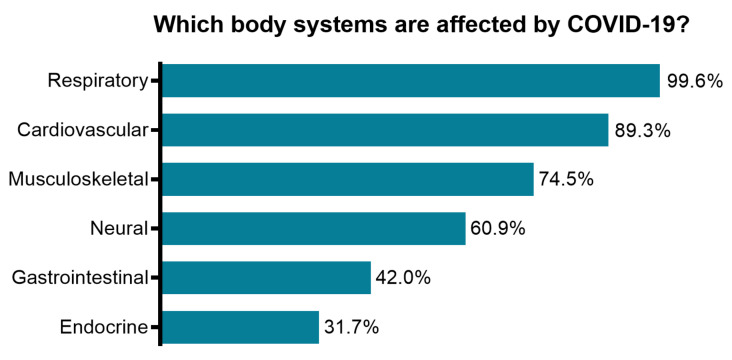
Current perception of survey participants on the potential impairment of body systems caused by COVID-19 infection.

Our data collection (December 2020–February 2021) was performed during the strong second wave of COVID-19 in Austria [1] The second infection wave (a peak at 9191 positively tested persons per day in November 2020) was 9 times higher than the first one at the end of March 2020. At this time several publications showed that in addition to the strong impairment of the respiratory organs, the SARS-CoV-2 virus could strongly affect other body systems [35,36]. To estimate the level of information regarding the clinical presentation of post-COVID-19 patients, survey participants were asked to state which body systems might be affected by the virus. In addition to 100% (*n* = 243) of the answers stating the respiratory system, most of the respondents mentioned the cardiovascular, musculoskeletal and neural systems (Figure 1).

**Figure 2:**
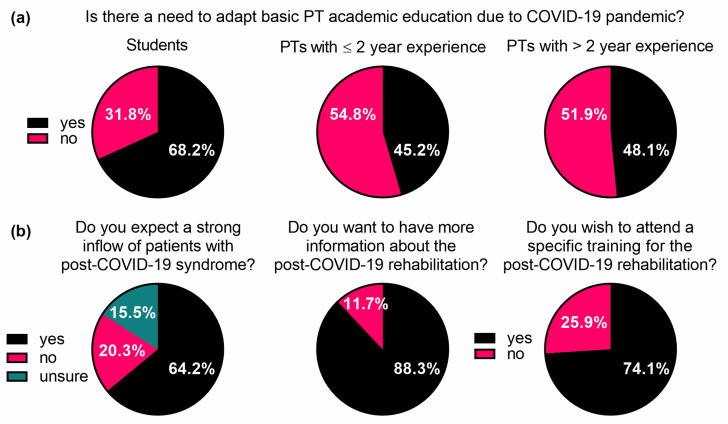
(**a**) Perception of physiotherapy students and physiotherapists regarding the adaptation of academic education due to the COVID-19 pandemic. (**b**) Assessment of participants’ expectations as to patient inflow as well as their interest in attending specific post-COVID-19 rehabilitation training. PT, physiotherapy; PTs, physiotherapists. See also Supplementary PDF 2.

The academic physiotherapy bachelor-level educational program in Austria is relatively new [37]; thus, we wanted to know if the COVID-19 pandemic situation was perceived as an important factor for bachelor program adaptations. Most of the physiotherapy students (68.2%, *n* = 44) favoured a COVID-19-specific adaptation in the basic academic education (Figure 2a). Stratified by work experience, 45.2% (*n* = 14) of the physiotherapists with less than two years of work experience and 48.1% (*n* = 74) of those with more than two years of work experience considered a COVID-19-specific adaptation of the basic academic program as necessary (Figure 2a). The following suggestions for an adaption were addressed with open questions: “Further information on COVID-19 including clinical presentation and treatment” (*n* = 56) and “Additional practical training in respiratory therapy techniques” (*n* = 44). Interestingly, the majority of respondents expected an increase in patient inflow with sequelae following COVID-19 infection (64.2%, *n* = 158); 20.3% (*n* = 50) did not expect a higher patient amount, and 15.5% (*n* = 38) were not sure (Figure 2b).

In addition, we assessed participants’ interest in obtaining COVID-19-related information and their intention to attend specific training for the rehabilitation of post-COVID-19 patients. The majority of respondents (88.3%, *n* = 212) stated that they would like to get more information about COVID-19 rehabilitation, and 74.1% (*n* = 172) indicated interest in attending specific training (Figure 2b). Only 11.2% (*n* = 27) of respondents reported feeling sufficiently informed about specific post-COVID-19 rehabilitation. A total of 23.7% (*n* = 57) stated that they felt rather well-informed, 27.8% (*n* = 67) did not feel sufficiently informed or even rated their information level as insufficient (37.3%, *n* = 90) (Table S1).

### 3.4. Physiotherapy Respiratory Rehabilitation of COVID-19 Survivors

Early reports from Italy (April–May 2020) [15] and the USA (March–June 2020) [38] demonstrated that COVID-19 can result in prolonged illness and persistent symptoms, targeting even young adults without underlying chronic medical conditions. Approximately a quarter of the survey respondents (28.3%, *n* = 69) had already received inquiries from patients with sequelae after a COVID-19 infection by February 2021 (end of our data collection). Most of the physiotherapists who received inquiries indicated having been working with post-COVID-19 patients (79.7%, *n* = 55). To estimate how experience with post-COVID-19 patients may influence the perception of specific examination and therapy, we included this factor in our survey evaluations.

### 3.5. Practical Experience: Physiotherapeutic Examination

Respiratory organs are the most affected by the SARS-COV-2 virus. Therefore, international and Austrian guidelines strongly focus on physiotherapeutic respiratory rehabilitation. To estimate the level of experience in outpatient settings, we asked the survey participants to rate their general experience in respiratory therapy using a 4-point Likert scale or ticking “no experience”. A total of 8.7% of the participants (*n* = 18) reported having no previous experience in respiratory physiotherapy, and 31.3% rated their experience as insufficient (*n* = 65). Participants who had no experience with certain examination and treatment techniques were excluded from the survey (*n* = 93). Respondents who reported prior experience (*n* = 125) rated it as very good (15.2%, *n* = 19), good (31.2%, *n* = 39) or at least sufficient (53.6%, *n* = 67).

**Figure 3:**
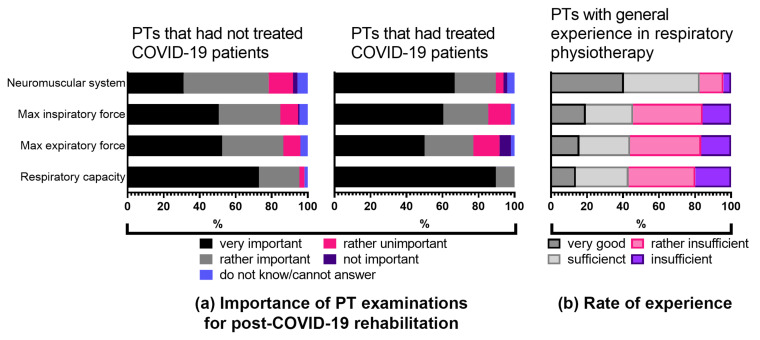
(**a**) Perception of physiotherapists regarding the importance of specific tests for examining post-COVID-19 patients. (**b**) Assessment of experience in performing specific tests by physiotherapists with previous experience in respiratory rehabilitation. PT, physiotherapy; PTs, physiotherapists. See also Supplementary PDF 2.

We further assessed the perception of physiotherapists regarding the importance of physiotherapeutic examination techniques for post-COVID-19 rehabilitation and evaluated practical experience in performing these tests as a part of respiratory rehabilitation (Figure 3a,b). Data were divided into two groups: physiotherapists currently treating patients with sequelae after COVID-19 infection (*n* = 48) and physiotherapists that had not treated post-COVID-19 patients so far (*n* = 170). The assessment of questions concerning the examination techniques consisted of four subthemes: examining the neuromusculoskeletal system (answered by *n* = 206), testing inspiratory (answered by *n* = 209) and expiratory (answered by *n* = 210) maximal force and testing respiratory capacity (answered by *n* = 215) (Figure 3a). The level of experience was rated from “very good” to “insufficient” (Figure 3b). A total of 72.9% (*n* = 124) of those therapists who had not treated COVID-19 patients so far and 89.6% (*n* = 43) of those that had already treated COVID-19 patients considered the testing of respiratory capacity as very important, but only 13.8% (*n* = 16) of the surveyed therapists rated their experience in the testing of respiratory capacity as very good. More than half of them considered their level of experience in testing respiratory capacity as rather insufficient or even insufficient (37.1% and 19.8%, respectively). The assessment of the importance of testing maximal inspiratory and expiratory forces compared with the results of experience in these areas are similar. In each case, more than half of the participants considered the examination techniques as very important, while only 15–20% reported a very good experience level (Figure 3a,b). Thus, those therapists who rated their general experience in respiratory physiotherapy as sufficient largely indicated a lack of experience when asked specifically about their experience in particular examination techniques (Figure 3b). Only in the area of neuromusculoskeletal examinations did the majority of respondents indicate very good or sufficient experience (Figure 3a,b). Concerning the assessment of the importance of testing the respiratory system, it is also noticeable that about twice as many therapists who had not worked with COVID-19 patients yet ticked “do not know/cannot answer” compared with those therapists who had already worked with COVID-19 patients (Figure 3a).

### 3.6. Practical Experience: Physiotherapeutic Treatment

**Figure 4:**
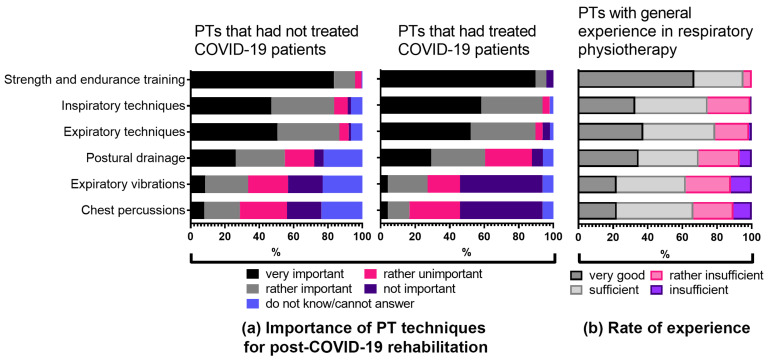
(**a**) Perception of physiotherapists regarding the importance of specific treatment techniques for post-COVID-19 rehabilitation. (**b**) Assessment of experience in specific treatment techniques by physiotherapists with previous experience in respiratory rehabilitation. PT, physiotherapy; PTs, physiotherapists. See also Supplementary PDF 2.

Next, we evaluated the perception of physiotherapists regarding the importance of specific physiotherapeutic treatment techniques for post-COVID-19 patients and asked them to rate their experiences in those specific treatment techniques (Figure 4a,b). The survey part dedicated to physiotherapeutic treatment techniques consisted of the following subthemes: a general strength and endurance training (answered by *n* = 211), respiratory techniques (inspiratory and expiratory, *n* = 200) and passive techniques including postural drainage (*n* = 172), expiratory vibration (answered by *n* = 171) and chest percussions (answered by *n* = 170) (Figure 4a). Again, data were divided into two groups: physiotherapists currently treating patients with sequelae after COVID-19 infection (*n* = 48) and physiotherapists that had not treated post-COVID-19 patients so far (*n* = 164). The level of experience in respiratory rehabilitation was rated from “very good” to “insufficient” (Figure 4b). Both therapists with and without previous COVID-19 patient experience considered general strength and endurance training as very important (89.6% (*n* = 43) and 83.5% (*n* = 137) accordingly) (Figure 4a). Respiratory techniques were rated as very important by 50% of physiotherapists without and 60% with post-COVID-19 patient experience. Interestingly, there was less confidence in assessing the importance of specific techniques in the area of passive physiotherapy interventions. Around 23% (*n* = 28) of therapists without post-COVID-19 patient experience answered “do not know/cannot answer” regarding the importance of passive physiotherapy techniques in the context of treating patients with sequelae after COVID-19.

In addition, it is notable that therapists with no experience rated the performance of passive hands-on techniques, such as expiratory vibrations and chest percussions, as almost twice as important as their colleagues who were already working with post-COVID-19 patients (Figure 4a). When we asked to rate the experience in performing specific techniques, of the therapists with previous experience in performing respiratory therapy (*n* = 125), 37.4% considered their experience in the performance of expiratory and 32.8% in inspiratory techniques as very good (Figure 4b), while up to a quarter of respondents rated their experience as “rather insufficient” or “insufficient” (Figure 4b, Table S2).

### 3.7. Device-Supported Respiratory Physiotherapy

**Figure 5:**
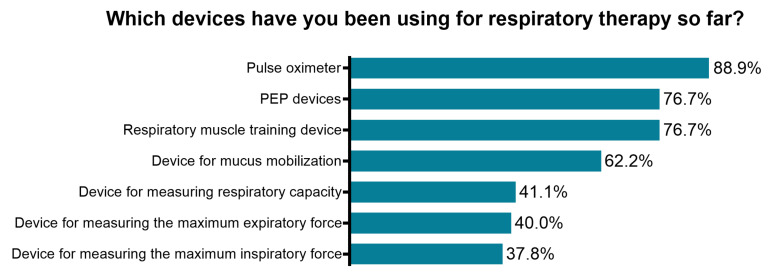
Assessment of the usage of respiratory rehabilitation devices/equipment among physiotherapists with previous experience in respiratory therapy (*n*= 90). PEP, positive expiratory pressure.

Using devices to assist respiratory measurements and therapy has clear advantages, such as visual feedback and the possibility to regulate exercise intensity. Therefore, device-supported rehabilitation is indispensable for effective respiratory therapy. The next survey section was developed to evaluate the current use of equipment related to respiratory rehabilitation. More than half of respondents stated that they had experience in using technical aids for respiratory physiotherapy (57.8%, *n* = 108). However, only 27.8% (*n* = 52) reported currently using technical aids in their practice. Further, physiotherapists were asked to specify which devices they already used in their therapy (Figure 5). Those respondents who had not yet used technical aids in the course of practicing respiratory rehabilitation (*n* = 79) stated that they had either too little experience in their application (38.0%, *n* = 30), had no aids available in their current work environment (68.4%, *n* = 54) or regarded the use of technical aids as unnecessary (2.5%, *n* = 2) (Supplementary Table S3).

## 4. Discussion

The COVID-19 pandemic has already altered physiotherapeutic practices and education formats. Growing knowledge about the acute and post-acute rehabilitation needs of COVID-19 patients will further affect healthcare systems and change educational curricular programs. It is expected that patients with long-term complications of this illness will potentially dominate medical practices for the next years, and rehabilitation professionals should be educated to provide care for the affected population [8].

To the authors’ knowledge, this is the first study to investigate perceptions, knowledge and experience of physiotherapists and physiotherapy students regarding post-acute COVID-19 rehabilitation. In a convenience sample of 203 physiotherapists working in outpatient settings, the majority recognized the importance of and wished to get more information about rehabilitation of post-COVID-19 patients, indicated an interest in additional training courses and expected an increased inflow of people with post-COVID-19 rehabilitation needs. At the time of our data collection, the Austrian physiotherapy association had already issued a first guideline for the rehabilitation of COVID-19, and several international scientific reports regarding respiratory rehabilitation were available [21,22,25,28]. Our results identify the lack of knowledge transfer as a possible barrier to the rapid implementation of post-COVID-19 rehabilitation into outpatient practices. These data indirectly support previous studies from Austria that show a low level of research activity among physiotherapists in Austria and insufficient knowledge translation of evidence-based findings into practice [39,40].

Among 49 final-year physiotherapy students, two-thirds acknowledged the urgent need to adapt basic academic educational curricula due to the current pandemic. The extent and type of future curricula changes remain to be defined. Current research data address major challenges for classical physiotherapeutic and chiropractic educational programs in a pandemic situation, including novel online models and virtual tools for manual therapy training [41,42].

In our study, we also described the perception of specific physiotherapeutic examination procedures and treatments for post-COVID-19 rehabilitation for the first time. It was observed before that survivors of severe acute respiratory syndrome, including SARS and MERS, exhibit persistent symptoms for at least a year post-recovery [43,44]. Patients that required admission to the ICU, mechanical ventilation, prolonged bed rest and immobilization may suffer from reduced muscle strength, metabolic alterations, impaired respiratory function, swallowing problems and cognitive and communication decay similar to post-intensive care syndrome [45,46,47]. Additionally, patients who had not been admitted to the ICU and/or were not hospitalized may suffer from post-acute COVID-19 syndrome due to virus-specific pathological changes, immunological reactions and inflammatory damage in response to SARS-Cov-2 (reviewed in [47]). Rehabilitation needs may further be elaborated by older age and comorbidities.

A spectrum of pulmonary manifestations ranging from dyspnoea to fibrotic lung damage in COVID-19 survivors was reported to last for up to 6 months post-infection [14,16]. Respiratory rehabilitation, also known as pulmonary rehabilitation, is generally recommended as a main rehabilitation strategy for patients with persistent respiratory symptoms [8,9,48], although clinical studies in patients with COVID-19 are still ongoing [14]. In addition to pulmonary impairments, COVID-19 survivors often suffer from neuromuscular complications, muscle weakness, fatigue and joint problems too. Thus, neuromuscular rehabilitation is likewise of great importance in the outpatient physiotherapeutic practice [7,8,26,48]. Participants of our study estimated the importance of different rehabilitation aspects for post-COVID-19 patients, reported about their experiences in neuromuscular rehabilitation and evaluated several aspects of respiratory rehabilitation. Most respondents rated their experience in neuromuscular rehabilitation as sufficient. Considering respiratory rehabilitation—even though physiotherapists have been trained and assessed on the theory and application of respiratory examination and treatment techniques as a part of their entry-level qualifications—most of the survey respondents did not rate their experience to be sufficient for the rehabilitation of patients suffering from post-COVID-19 syndrome. The comparison of respondents who had already treated COVID-19 patients with other physiotherapists revealed that both groups endorsed the importance of maximal inspiratory and expiratory force examination and testing of respiratory capacity to the same extent, while the “post-COVID-19-experienced” therapists acknowledged the importance of the neuromuscular system to a larger extent. This difference reflects the current general knowledge about the SARS-CoV-2 virus affecting respiratory organs and is somewhat a restricted view of physiotherapists on other affected body systems. In line with this, all survey respondents indicated the respiratory system as being affected by the virus, while fewer respondents indicated neural and musculoskeletal system involvement (61% and 75%, accordingly).

Devices to measure respiratory parameters and assist the training of respiratory muscles are available, and several studies have shown the beneficial effect of respiratory muscle training in asthma and COPD [49,50,51]. Respondents of our study reported a lack of technical resources to enable efficient respiratory rehabilitation and a paucity of practical experience in using supporting devices. Deficits were especially reported in measuring respiratory parameters, including respiratory capacity, maximal inspiratory and expiratory force.

All over the world, the percentage of COVID-19 survivors developing post-COVID-19 syndrome is growing even beyond the initially estimated 10–20% [52]. To the best of our knowledge, no reports have summarized available resources in the rehabilitation section so far. It can be estimated that the post-COVID-19 patient influx will overwhelm existing rehabilitation facilities. In Austria, only 22 out of 126 rehabilitation facilities are equipped to perform specific respiratory rehabilitation [53]. In the long run, the COVID-19 pandemic is forcing changes in several domains: current curricula, new educational programs with a focus on interdisciplinary rehabilitation, advanced training for professionals as well as the extension of existing rehabilitation facilities. However, the ongoing pandemic situation dictates rapid adaptation of patient management, with increased patient outsourcing to the extramural sector. Currently, there are around 8000 self-employed physiotherapists in Austria with a prevalent specialization in orthopaedics/traumatology and neurology [34,54]. Taking into account the willingness to participate in COVID-19-specific education that we found in our study, a prompt offering of advanced training would provide an efficient way of addressing post-COVID-19 rehabilitation needs.

### Limitations

In terms of age, gender and work specialization distribution, the sample of participants was similar to previous studies conducted in Austria [23,55]. The official registry of Austrian physiotherapists lists around 16,000 persons, half of them being self-employed therapists [34]. However, the registry does not provide information about the percentage of therapists working in in- or outpatient settings. The exact number of physiotherapists in the study target group remains unclear; thus, no exact response rate can be calculated. Additional limitations concern therapists with a personal interest in COVID-19 who might have been more likely to participate and might have had better knowledge than non-respondents. Study limitations also include the chosen online setting and the associated absence of a researcher; possible misunderstandings of the survey items could not be identified or clarified. Further risks of bias in our study concern the recruitment strategy for participants through digital media and the designed online survey. Our study may have excluded physiotherapists with lower technical affinity. Concerning physiotherapy students, although the sampling pool had regional representation of nine Austrian provinces, it did not include students from all physiotherapy programs in Austria, which may affect the generalizability of our results.

Due to the aforementioned limitations and the sample size, generalized conclusions referring to the population of Austrian physiotherapists should be drawn with care. Nevertheless, this is, to date, the only survey capturing the perception of more than 250 therapists and students in a variety of physiotherapeutic specializations.

## 5. Conclusions

The rehabilitation needs of COVID-19 survivors are increasingly recognized. Physiotherapeutic interventions are especially relevant to combat respiratory and neuromuscular dysfunctions in these patients. Our study showed that physiotherapists and physiotherapy students in Austria acknowledge the requirement of quick changes in academic education and further professional development. Data presented here highlight a clear gap between upcoming rehabilitation needs and the current capabilities of physiotherapists in Austria. Our results suggest that improving knowledge transfer and providing technical devices will accelerate the implementation of effective post-acute COVID-19 rehabilitation into outpatient physiotherapy practice. There is an urgent need to develop new education and training programs with a focus on the interdisciplinary rehabilitation of patients with the post-COVID-19 syndrome. The evaluation of these programs should become an important priority for future international studies.

## Figures and Tables

**Table 1:** Participant characteristics.

Respondents	*n*	%	Mdn	IQR
Gender	255			
Female	193	75.7		
Male	61	23.9		
Not specified	1	0.4		
Qualification	255			
Undergraduate				
In education	49	19.2		
Diploma	71	27.8		
Bachelor	82	32.2		
Postgraduate				
Master or equivalent	50	19.6		
Doctoral degree	3	1.2		
Work experience ^b^	203		10.0	14.0
≤2 years	34	16.7	1.0	2.0
>2 years	169	83.3	12.0	13.0
Current workplace ^ab^ (251 total responses)	203			
Independent physiotherapy practice	139	68.7		
Outpatient rehabilitation	28	13.7		
Outpatient clinic (public hospital)	25	12.3		
Outpatient clinic (private hospital)	12	5.9		
Other	47	23.2		
Clinical speciality ^ab^ (431 total responses)	203			
Orthopaedics	141	69.5		
Traumatology	101	49.8		
Neurology	45	22.2		
Internal medicine	44	21.7		
Geriatrics	38	18.7		
Paediatrics	22	10.8		
Gynaecology	12	5.9		
Other	28	13.8		

Abbreviations: *n* (sample size); % (percentage); Mdn (median); IQR (interquartile range). ^a^ Multiple responses possible; ^b^ excluding students.

## Data Availability

All data used in the analysis are available upon request to the corresponding author.

## Supplementary Materials

The following are available online at https://www.mdpi.com/article/10.3390/ijerph18168730/s1, Table S1: Assessment of participants’ information status about the specific post-COVID-19 rehabilitation, Table S2: Assessment of experience in specific treatment techniques by physiotherapists with previous experience in respiratory rehabilitation, Table S3: Participants reasons for not using technical aids. Supplementary PDF 1: Questionnaire in English Supplementary PDF 2: Data for Figures 2, 3 and 4.
