# Supplementary material for: Post-COVID-19 Rehabilitation: Perception and Experience of Austrian Physiotherapists and Physiotherapy Students
Source: Int J Environ Res Public Health. 2021 Aug 18;18(16):8730. doi: 10.3390/ijerph18168730 (PMC8394152; doi:10.3390/ijerph18168730)
Supplement: Supplementary file 1 [file ijerph-18-08730-s001.zip › Supplementary PDF 1.pdf]

Dear physiotherapists, dear colleagues,

the COVID-19 pandemic poses great challenges to all of us in our daily work as physiotherapists. The aim of this research project is to assess the feasibility of effective rehabilitation for patients with sequelae of COVID-19 infection in an outpatient physiotherapy setting.

We would like to ask for your help by sharing your opinion, experience and perception by filling in the questionnaire we have prepared.

The completion of this questionnaire will take 5-10 min.

All data will be collected anonymously, cannot be attributed to any individual and will be used exclusively for this research project.

By submitting the completed questionnaire, the participant agrees to take part in the study. A subsequent withdrawal of the declaration of consent is not possible due to the anonymous survey procedure.

Thank you very much for your willingness to complete this questionnaire!

FH Gesundheit Tirol/Health University of Applied Sciences Tyrol
Innrain 98
A-6020 Innsbruck, Austria
Correspondence: Natalia Schiefermeier-Mach, PhD
natalia.schiefermeier-mach@fhg-tirol.ac.at (+43 512 5322 75482)

Part 1: Demographic data

1. What is your gender?

____

2. What is your age in years?

____

Part 2: Information on your education and current field of work

1. What is your highest completed, professionally relevant education?

□ In education
□ Diploma
□ Bachelor
□ Master or equivalent
□ Doctoral degree

2. How many years of professional experience do you have as a physiotherapist?

____

3. In which of the following areas do you currently work? (Multiple answers possible)

□ Outpatient clinic (public hospital)
□ Outpatient clinic (private hospital)
□ Outpatient rehabilitation
□ Independent physiotherapy practice
□ Other: _____________

4. In which areas did you specialize? (Multiple answers possible)

□ Internal medicine
□ Neurology
□ Orthopaedics
□ Traumatology
□ Geriatrics
□ Pediatrics
□ Gynaecology
□ Other:______________

Part 3: Covid-19 specific questions

1. Which body systems do you think might be affected by a COVID-19 infection? (Multiple answers possible)

□ Respiratory system
□ Cardiovascular system
□ Endocrine system
□ Gastrointestinal system
□ Musculoskeletal system
□ Nervous system
□ Other: ______________

2. Is there a need to adapt basic physiotherapeutic academic education due to COVID-19 pandemic?

□ Yes
□ No

3. Please indicate your proposed change for the basic academic physiotherapy training.

____

4. Do you expect a strong inflow of patients with post-COVID-19 syndrome?

□ Yes
□ No
□ I don’t know.

5. Do you want to have more information about the post-COVID-19 rehabilitation?

□ Yes
□ No

6. Do you wish to attend a specific training for the post-COVID-19 rehabilitation?

□ Yes
□ No

7. How much do you agree with the following statement?
I feel adequately informed regarding the physiotherapeutic rehabilitation of patients with sequelae after a COVID-19 infection.

true	rather true	rather not true	not true
□	□	□	□

8. Have you had any requests for respiratory rehabilitation for patients with sequelae after a COVID-19 infection?

□ Yes
□ No

9. Do you currently work, or have you been working with patients with sequelae after a COVID-19 infection?

- ☐ Yes
- ☐ No

Part 4: Questions about respiratory physiotherapy experience

1. How would you rate your experience in respiratory physiotherapy?

very good	good	sufficient	insufficient	no experience
☐	☐	☐	☐	☐

2. How important do you consider the **testing of the neuromusculoskeletal system** for examining patients with sequelae after COVID-19 infection?

very important	rather important	rather unimportant	not important	do not know/ cannot answer
☐	☐	☐	☐	☐

3. How important do you consider the **testing of the maximum inspiratory force** for examining patients with sequelae after COVID-19 infection?

very important	rather important	rather unimportant	not important	do not know/ cannot answer
☐	☐	☐	☐	☐

4. How important do you consider the **testing of the maximum expiratory force** for examining patients with sequelae after COVID-19 infection?

very important	rather important	rather unimportant	not important	do not know/ cannot answer
☐	☐	☐	☐	☐

5. How important do you consider the **testing of the respiratory capacity** for examining patients with sequelae after COVID-19 infection?

very important	rather important	rather unimportant	not important	do not know/ cannot answer
☐	☐	☐	☐	☐

6. How do you rate your experience in **testing the neuromuscular system**?

very good	sufficient	rather sufficient	insufficient
☐	☐	☐	☐

7. How do you rate your experience in **testing the inspiratory maximum force**?

very good	sufficient	rather sufficient	insufficient
☐	☐	☐	☐

8. How do you rate your experience in **testing the expiratory maximum force**?

very good	sufficient	rather sufficient	insufficient
☐	☐	☐	☐

9. How do you rate your experience in **testing the respiratory capacity**?

very good	sufficient	rather sufficient	insufficient
☐	☐	☐	☐

10. How important do you consider the use of **strength and endurance training** to treat patients with sequelae after a COVID-19 infection?

very important	rather important	rather unimportant	not important	do not know/ cannot answer
☐	☐	☐	☐	☐

11. How important do you consider the use of **inspiratory techniques** to treat patients with sequelae after a COVID-19 infection?

very important	rather important	rather unimportant	not important	do not know/ cannot answer
☐	☐	☐	☐	☐

12. How important do you consider the use of **expiratory techniques** to treat patients with sequelae after a COVID-19 infection?

very important	rather important	rather unimportant	not important	do not know/ cannot answer
☐	☐	☐	☐	☐

13. How important do you consider the use of **postural drains** to treat patients with sequelae after a COVID-19 infection?

very important	rather important	rather unimportant	not important	do not know/ cannot answer
☐	☐	☐	☐	☐

14. How important do you consider the use of **vibration techniques** to treat patients with sequelae after a COVID-19 infection?

very important	rather important	rather unimportant	not important	do not know/ cannot answer
☐	☐	☐	☐	☐

15. How important do you consider the use of **chest percussions** to treat patients with sequelae after a COVID-19 infection?

very important	rather important	rather unimportant	not important	do not know/ cannot answer
☐	☐	☐	☐	☐

16. How would you rate your experience in performing a **strength and endurance training**?

very good	sufficient	rather sufficient	insufficient
□	□	□	□

17. How would you rate your experience in performing **inspiratory techniques**?

very good	sufficient	rather sufficient	insufficient
□	□	□	□

18. How would you rate your experience in performing **expiratory techniques**?

very good	sufficient	rather sufficient	insufficient
□	□	□	□

19. How would you rate your experience in performing **postural drains**?

very good	sufficient	rather sufficient	insufficient
□	□	□	□

20. How would you rate your experience in performing **vibration techniques**?

very good	sufficient	rather sufficient	insufficient
□	□	□	□

21. How would you rate your experience in performing **chest percussions**?

very good	sufficient	rather sufficient	insufficient
□	□	□	□

22. Have you already used technical aids for specific respiratory therapy?

□ Yes
□ No

23. In your current work environment, do you have the possibility to use technical aids for the respiratory therapy for patients with sequelae of Covid-19 infection?

□ Yes
□ No

24. Which devices have you been using for respiratory therapy so far? (Multiple answers possible)

□ Pulse oximeter
□ PEP devices
□ Respiratory muscle training device
□ Device for mucus mobilization
□ Device for measuring respiratory capacity
□ Device for measuring the maximum expiratory force
□ Device for measuring the maximum inspiratory force

25. Why have you refrained from using technical aids so far? (Multiple answers possible)

□ I have too little experience in the application
□ I do not find the use of technical aids necessary.
□ No technical aids are available at current work environment.
