# Supplementary material for: Post-COVID-19 Rehabilitation: Perception and Experience of Austrian Physiotherapists and Physiotherapy Students
Source: Int J Environ Res Public Health. 2021 Aug 18;18(16):8730. doi: 10.3390/ijerph18168730 (PMC8394152; doi:10.3390/ijerph18168730)
Supplement: Supplementary file 1 [file ijerph-18-08730-s001.zip › Supplementary PDF 2.pdf]

Data for Figure 2a

Is there a need to adapt basic physiotherapeutic academic education due to COVID-19 pandemic?

		yes		no	
		n	(%)	n	(%)
Students	n=44	30	(61.2)	14	(31.8)
PTs with ≤ 2 years experience	n=31	14	(45.2)	17	(54.8)
PTs with > 2 years experience	n=154	74	(48.1)	80	(51.9)
Total	n=229	118	(51.5)	111	(48.5)

Data for Figure 2b

Assessment of participants' expectations, interest and wish to attend to specific post-COVID-19 rehabilitation trainings

		yes		no		unsure	
		n	(%)	n	(%)	n	(%)
**1) Do you expect a strong inflow of patients with post-COVID-19 syndrome?**	n=246	158	(64.2)	50	(20.3)	38	(15.5)
**2) Do you want to have more information about the post-COVID-19 rehabilitation?**	n=240	212	(88.3)	28	(11.7)	0	(0.0)
**3) Do you wish to attend a specific training for the post-COVID-19 rehabilitation?**	n=232	172	(74.1)	60	(25.9)	0	(0.0)

Data for Figure 3a

How important do you consider the testing of the ... for examining patients with sequelae after COVID-19 infection?

n=170 without patients (a) n=48 with patients (b)	very important				rather important				rather unimportant				not important				do not know/ cannot answer			
	a		b		a		b		a		b		a		b		a		b	
	n	(%)	n	(%)	n	(%)	n	(%)	n	(%)	n	(%)	n	(%)	n	(%)	n	(%)	n	(%)
Neuromuscular system	53	(31.2)	32	(66.7)	80	(47.1)	11	(22.9)	23	(13.5)	2	(4.2)	4	(2.4)	1	(2.1)	10	(5.9)	2	(4.2)
Max inspiratory force	86	(50.6)	29	(60.4)	58	(34.1)	12	(25.0)	17	(10.0)	6	(12.5)	1	(0.6)	0	(0.0)	8	(4.7)	1	(2.1)
Max expiratory force	89	(52.4)	24	(50.0)	58	(34.1)	13	(27.1)	16	(9.4)	7	(14.6)	0	(0.0)	3	(6.3)	7	(4.1)	1	(2.1)
Respiratory capacity	124	(72.9)	43	(89.6)	38	(22.4)	5	(10.4)	5	(2.9)	0	(0.0)	0	(0.0)	0	(0.0)	3	(1.8)	0	(0.0)

Data for Figure 3b

How do you rate your experience in testing the ...?

n=124 PTs with general experience	very good		sufficient		rather insufficient		insufficient	
in respiratory therapy	n	(%)	n	(%)	n	(%)	n	(%)
1) Neuromuscular system	49	(40.5)	51	(42.1)	16	(13.2)	5	(4.1)
2) Max inspiratory force	22	(19.3)	30	(26.3)	44	(38.6)	18	(15.8)
3) Max expiratory force	18	(15.8)	32	(28.1)	45	(39.5)	19	(16.7)
4) Respiratory capacity	16	(13.8)	34	(29.3)	43	(37.1)	23	(19.8)

Data for Figure 4a

How important do you consider the use of ... to treat patients with sequelae after COVID-19 infection?

n=164 without patients (a)<br>n=48 with patients (b)	very important				rather important				rather unimportant				not important				do not know/ cannot answer			
	a		b		a		b		a		b		a		b		a		b	
	n	(%)	n	(%)	n	(%)	n	(%)	n	(%)	n	(%)	n	(%)	n	(%)	n	(%)	n	(%)
1) Strength and endurance training	137	(83.5)	43	(89.6)	20	(12.2)	3	(6.3)	6	(3.7)	2	(4.2)	0	(0.0)	0	(0.0)	1	(0.6)	0	(0.0)
2) Inspiratory techniques	77	(47.0)	28	(58.3)	60	(36.6)	17	(35.4)	13	(7.9)	2	(4.2)	3	(1.8)	0	(0.0)	11	(6.7)	1	(2.1)
3) Expiratory techniques	83	(50.6)	25	(52.1)	59	(36.0)	18	(37.5)	9	(5.5)	2	(4.2)	2	(1.2)	2	(4.2)	11	(6.7)	1	(2.1)
4) Postural drainage	43	(26.2)	14	(29.2)	47	(28.7)	15	(31.3)	28	(17.1)	13	(27.1)	9	(5.5)	3	(6.3)	37	(22.6)	3	(6.3)
5) Expiratory vibrations	14	(8.5)	2	(4.2)	41	(25.0)	11	(22.9)	38	(23.2)	9	(18.8)	33	(20.1)	23	(47.9)	38	(23.2)	3	(6.3)
6) Chest percussions	13	(7.9)	2	(4.2)	34	(20.7)	6	(12.5)	45	(27.4)	14	(29.2)	33	(20.1)	23	(47.9)	39	(23.8)	3	(6.3)

Data for Figure 4b

How do you rate your experience in performing ...?

n=124 PTs with general experience	very good		sufficient		rather insufficient		insufficient	
in respiratory therapy	n	(%)	n	(%)	n	(%)	n	(%)
1) Strength and endurance training	83	(66.9)	35	(28.2)	6	(4.8)	0	(0.0)
2) Inspiratory techniques	40	(32.8)	51	(41.8)	30	(24.6)	1	(0.8)
3) Expiratory techniques	46	(37.4)	51	(41.5)	24	(19.5)	2	(1.6)
4) Postural drainage	41	(34.7)	41	(34.7)	28	(23.7)	8	(6.8)
5) Expiratory vibrations	22	(22.0)	40	(40.0)	26	(26.0)	12	(12.0)
6) Chest percussions	23	(22.1)	46	(44.2)	24	(23.1)	11	(10.6)
