# Supplementary material for: Post-COVID-19 Rehabilitation: Perception and Experience of Austrian Physiotherapists and Physiotherapy Students
Source: Int J Environ Res Public Health. 2021 Aug 18;18(16):8730. doi: 10.3390/ijerph18168730 (PMC8394152; doi:10.3390/ijerph18168730)
Supplement: Supplementary file 1 [file ijerph-18-08730-s001.zip › Supplementary Table_S1.pdf]

Table S1. Assessment of participants' information status about the specific post-COVID-19 rehabilitation

**Physiotherapists information status** n=241	**sufficient**		**rather sufficient**		**rather insufficient**		**insufficient**	
	n	(%)	n	(%)	n	(%)	n	(%)
	27	(11.2)	57	(23.7)	67	(27.8)	90	(37.3)
