# Supplementary material for: Post-COVID-19 Rehabilitation: Perception and Experience of Austrian Physiotherapists and Physiotherapy Students
Source: Int J Environ Res Public Health. 2021 Aug 18;18(16):8730. doi: 10.3390/ijerph18168730 (PMC8394152; doi:10.3390/ijerph18168730)
Supplement: Supplementary file 1 [file ijerph-18-08730-s001.zip › Supplementary Table_S2.pdf]

Table S2. Assessment of experience in specific treatment techniques by physiotherapists with previous experience in respiratory rehabilitation

**PTs with general experience in respiratory therapy** n=124	**very good**		**sufficient**		**rather insufficient**		**insufficient**	
	n	(%)	n	(%)	n	(%)	n	(%)
Strength and endurance training	83	(66.9)	35	(28.2)	6	(4.8)	0	(0.0)
Inspiratory techniques	40	(32.8)	51	(41.8)	30	(24.6)	1	(0.8)
Expiratory techniques	46	(37.4)	51	(41.5)	24	(19.5)	2	(1.6)
Postural drainage	41	(34.7)	41	(34.7)	28	(23.7)	8	(6.8)
Expiratory vibrations	22	(22.0)	40	(40.0)	26	(26.0)	12	(12.0)
Chest percussions	23	(22.1)	46	(44.2)	24	(23.1)	11	(10.6)
