# Supplementary material for: Post-COVID-19 Rehabilitation: Perception and Experience of Austrian Physiotherapists and Physiotherapy Students
Source: Int J Environ Res Public Health. 2021 Aug 18;18(16):8730. doi: 10.3390/ijerph18168730 (PMC8394152; doi:10.3390/ijerph18168730)
Supplement: Supplementary file 1 [file ijerph-18-08730-s001.zip › Supplementary Table_S3.pdf]

Table S3. Participants reasons for not using technical aids

n=86	n	(%)
too little experience	30	(38.0)
no aids available at current work environment	54	(68.4)
use is not necessary	2	(2.5)
